# Controlled multistep synthesis in a three-phase droplet reactor

**DOI:** 10.1038/ncomms4777

**Published:** 2014-05-06

**Authors:** Adrian M. Nightingale, Thomas W. Phillips, James H. Bannock, John C. de Mello

**Affiliations:** 1 Department of Chemistry, Imperial College London, Exhibition Road, South Kensington, London SW7 2AY, UK; 2 Present address: National Oceanographic Centre, University of Southampton Waterfront Campus, European Way, Southampton SO14 3ZH, UK

## Abstract

Channel-fouling is a pervasive problem in continuous flow chemistry, causing poor product control and reactor failure. Droplet chemistry, in which the reaction mixture flows as discrete droplets inside an immiscible carrier liquid, prevents fouling by isolating the reaction from the channel walls. Unfortunately, the difficulty of controllably adding new reagents to an existing droplet stream has largely restricted droplet chemistry to simple reactions in which all reagents are supplied at the time of droplet formation. Here we describe an effective method for repeatedly adding controlled quantities of reagents to droplets. The reagents are injected into a multiphase fluid stream, comprising the carrier liquid, droplets of the reaction mixture and an inert gas that maintains a uniform droplet spacing and suppresses new droplet formation. The method, which is suited to many multistep reactions, is applied to a five-stage quantum dot synthesis wherein particle growth is sustained by repeatedly adding fresh feedstock.

## Abstract (web summary)

Droplet chemistry is less susceptible to channel-fouling than single-phase flow chemistry, but is largely limited to simple reactions where all reagents are preloaded into droplets. Here, the authors report a method for multistep chemistry in droplets, using two immiscible liquids and a gas to achieve controlled, sequential reagent addition.

## Graphical abstract

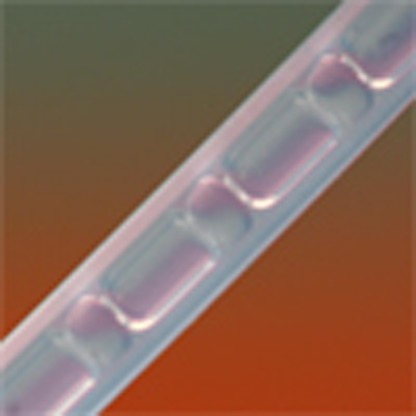

Microfluidic synthesis procedures can offer significant advantages over conventional batch methods in terms of improved product control, reproducibility and automation^1^. The majority of microfluidic research to date has involved single-phase flow reactors, in which miscible reagent streams are continuously injected into channels where they react to form the final product^2^. Recently, however, two-phase flow reactors have been attracting interest for applications where higher levels of reaction control and greater operational stability are required^3^,^4^. In two-phase reactors, an immiscible fluid—either a liquid or a gas—is injected into the channel alongside the reaction phase, causing one or both phases to divide into a succession of discrete, dimensionally confined slugs or droplets. Key advantages of two-phase flow over continuous flow include: enhanced mixing, superior synthetic control due to lower sample volumes, and reduced susceptibility to fouling.

Droplet flow is a specific form of two-phase flow^5^ that occurs when the immiscible fluid is a liquid that preferentially wets the channel walls, causing the reagent phase to divide into a train of discrete, near-identical low-volume droplets that travel through the channel at a common speed. Owing to their small (typically submicrolitre) volumes the droplets are extremely uniform with regards to chemical composition and temperature, and so provide a highly controlled environment for carrying out chemical reactions. Moreover, since the droplets are kept away from the channel walls by the inert ‘carrier’ liquid, the chance of reactor fouling due to precipitation of reactants or products on the channel wall is virtually eliminated, ensuring a stable, unchanging reaction environment.

Droplet-based flow reactors have proven to be of particular value for the controlled synthesis of functional materials whose properties are strongly influenced by the reaction conditions. For instance, significant improvements in product control (with respect to both batch and continuous flow synthesis) have been demonstrated for a broad range of optoelectronic materials including metal nanocrystals^6^,^7^,^8^, quantum dots^9^,^10^,^11^,^12^ and conjugated polymers^13^,^14^. To date, however, droplet synthesis has been largely limited to simple one-step procedures, in which all reagents are loaded into the droplets at the outset, greatly restricting the range of chemistries that can be employed. To properly exploit the benefits of droplet flow, and widen the palette of accessible chemistries/materials, there is an obvious need to develop multistep droplet reactors in which reagents can be sequentially added into the flowing droplets as the reaction proceeds.

There are very few reports of controlled multistep synthesis in droplet flow due to the difficulty of introducing quantitative amounts of reagent into a flowing droplet stream^15^. Two broad strategies for dosing droplets have been reported in the literature. In the first approach, droplet fusion, the new reagents are introduced into the flow reactor as a separate droplet stream, which is then merged on a pairwise basis with the original droplet stream (Fig. 1a). This can be achieved using special channel architectures to bring the droplets together^16^,^17^,^18^, sometimes assisted by the application of an electric field to lower the interfacial tension between droplets^19^,^20^,^21^. In the second approach, direct injection, the new reagent is injected into the channel as a continuous laminar stream that spontaneously adds to the droplets as they pass (Fig. 1b)^15^,^22^,^23^,^24^,^25^.

Since there is no need for specially fabricated channel structures or peripheral equipment, direct injection is much simpler to implement than droplet fusion and is therefore a preferable approach for multistep chemistry. In practice, however, it suffers from two key weaknesses. First, to achieve consistent dosing, a uniform spacing of droplets is required so that equal amounts of reagent accumulate at the delivery point prior to the arrival of each new droplet. Unfortunately, this is difficult to achieve in a two-phase system since local variations in channel height, diameter and temperature can introduce ‘jitter’ into the velocities of the individual droplets and thereby disrupt the uniform spacing. Second, the method is limited to the addition of reagent volumes that are small in relation to the droplet volume, since injecting reagents at excessively high flow rates results in the generation of new droplets of the injected reagent. Both issues introduce disorder into the dosing process and undermine the viability of using direct injection for controlled multistep chemistry.

There is consequently a need for new, robust and easy-to-implement dosing methods that can reliably deliver controlled quantities of reagent into flowing droplets without the need for sophisticated channel architectures or extraneous equipment. Here we show how a simple three-phase reactor, employing a gas and two immiscible liquids, can be used to ensure reproducible dosing by direct injection. We show how the inclusion of a gas phase in the fluid stream improves the reliability of direct injection by maintaining a uniform droplet spacing and suppressing the unwanted formation of new droplets. We further show how three-phase systems can enable multistep chemistry in droplets, using multistep quantum dot growth as an exemplar.

## Results

### Generation of a three-phase fluid stream

**Figure 2:**
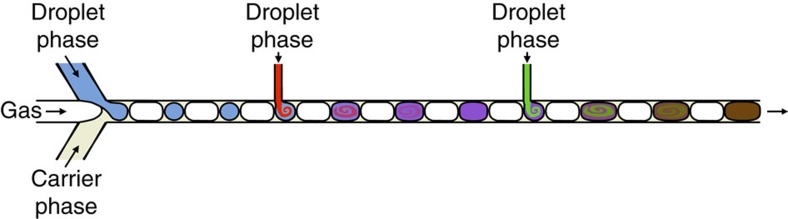
**Direct injection of reagents into a three-phase fluid stream**. Schematic showing the generation of a three-phase flow followed by direct injection of solvent into the droplets.

**Figure 3:**
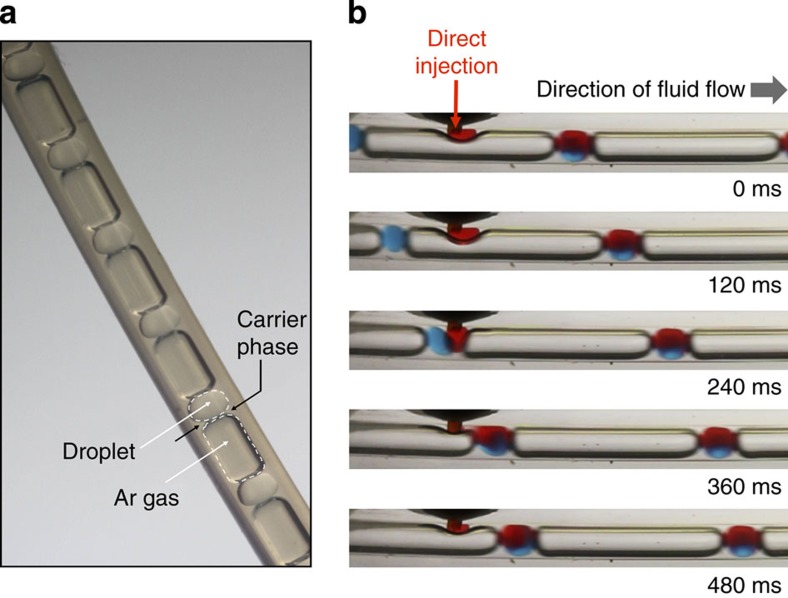
Images of three-phase fluid stream and process of reagent addition. (**a**) Photograph of a three-phase flow comprising octadecene droplets, argon gas bubbles and a perfluorinated polyether carrier fluid, passing through 1 mm inner diameter PTFE tubing. (**b**) A sequence of still images showing the direct injection of red-dyed octadecene into blue-dyed octadecene droplets, using Ar gas as a spacer and PFPE as the carrier fluid.

Three-phase fluid streams have previously been used in droplet microfluidics to prevent coalescence between adjacent droplets (with the gas phase acting as a simple spacer)^7^,^26^,^27^, but their use in downstream reagent addition (a requirement for all multistep syntheses) has not, to our knowledge, been previously reported. The general principle of our approach is shown in Fig. 2. A three-phase flow—comprising an organic or aqueous solvent, a fluorous carrier liquid and an inert spacer gas—is generated at the conjunction of a three-way mixer (see Methods) and passed into a polytetrafluoroethylene (PTFE) capillary. The volume of the carrier liquid is kept low to minimize the distance between the droplets and the gas phase, while still being sufficient to ensure complete isolation of the droplets from the channel walls. Figure 3a shows a photograph of a three-phase flow in a 1-mm inner diameter (ID) PTFE capillary, comprising an alternating steam of argon gas and octadecene (ODE) droplets dispersed in an immiscible perfluorinated polyether (PFPE) carrier liquid that preferentially wets the capillary wall. The highly ordered nature of the three-phase flow is clear, with the Ar gas maintaining a uniform spacing between the ODE droplets. (Note, the PFPE carrier liquid is somewhat difficult to discern in the image due to its low volume relative to the other components, but its presence close to the channel wall may be inferred from the boundaries of the other two components, which both curve away from the channel wall at the ODE/Ar interfaces.)

### Direct injection of reagents via a T-junction

The process of reagent addition is shown in the time-lapse images in Fig. 3b and in Supplementary Movie 1. Additional reagent (dissolved in the droplet-phase solvent) is continuously introduced into the flowing droplet stream at a T-junction, where it inserts into the existing droplets, causing them to grow in volume. The images and movie show a pregenerated three-phase ODE/Ar/PFPE flow, in which blue-dyed ODE droplets travel along a 1-mm diameter channel at a linear velocity of 11.4 mm s^−1^ and pass through a T-junction where a laminar stream of red-dyed ODE is injected. When a gas bubble is transiting the T-junction (for example, *t*=0, 120 ms), the red-dyed ODE accumulates at the outlet and continues to do so until a droplet arrives (*t*=240 ms), at which point it fuses with the droplet. The enlarged droplet is then carried downstream where mixing occurs. Repeated dosing of individual droplets may be achieved through the use of multiple T-junctions (as illustrated in Fig. 2).

The presence of the gas phase is critical to achieving controlled direct reagent addition for two principal reasons. First, the gas maintains a uniform droplet spacing, thereby ensuring each droplet receives the same dose of new reagent. Second, since it is the gas and not the carrier liquid that keeps the droplets apart, there is an insufficient volume of carrier liquid to allow the formation of new droplets. Therefore, the new solvent must add into the droplets or else it must form new droplets within the gas, with the latter process splitting the gas bubble in two. Splitting the gas bubbles results in much larger interfacial areas compared with injection into the droplets and is consequently disfavoured energetically.

### Comparison of reagent addition in two- and three-phase flows

**Figure 4:**
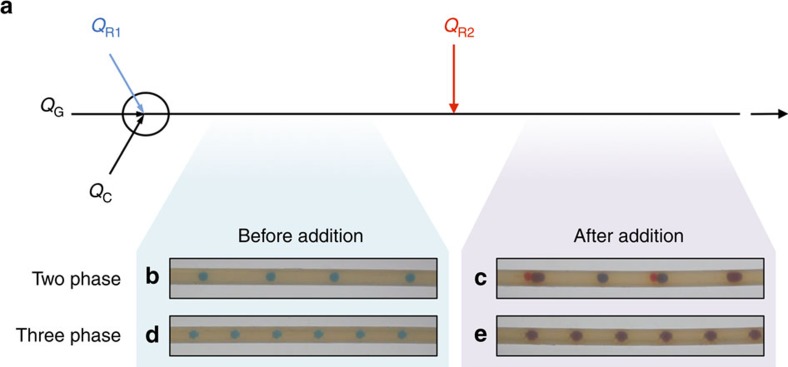
Images of two- and three-phase fluid streams before and after reagent addition. (**a**) Schematic showing experimental set-up used to observe the insertion of red-dyed solvent into blue droplets: a two- or three-phase stream of blue-dyed ODE droplets is first generated by flowing ODE/PFPE or ODE/PFPE/Ar through a PTFE conjunction, then a stream of red-dyed ODE (*Q*_R2_) is injected into the flowing droplets using a T-junction. (**b**–**e**) Representative images of the flowing droplets before and after solvent addition for the two- and three-phase flows.

To compare the efficacy of direct addition in two- and three-phase droplet flow, a simple visual test was carried out in which a continuous stream of red-dyed ODE was added to a droplet stream of blue-dyed ODE, and variations in the final colours and sizes of the droplets were used to evaluate the consistency of the dosing (Fig. 4). For the three-phase experiment, two syringe pumps were used to inject PFPE carrier liquid and blue-dyed ODE solvent into two inlets of a three-way junction at respective rates *Q*_C_ and *Q*_R1_, while a mass flow controller was used to inject argon gas into the third inlet at a rate *Q*_G_. A two-metre length of 1-mm-ID PTFE capillary was used to connect the outlet of the mixer to the main inlet of a T-junction (ID=1 mm, see Methods). Red-dyed ODE solvent was injected as a continuous stream from a third syringe pump into the side-inlet of the T-junction (ID=150 μm) at a rate *Q*_R2_, and a second piece of 1-mm-ID PTFE capillary was connected to the outlet of the T-junction. A colour digital single-lens reflex (DSLR) camera fitted with a 60-mm macro lens was used to record images of the flowing droplets before and after the T-junction. *Q*_R1_ and *Q*_R2_ were both set to 30 μl min^−1^, *Q*_C_ was set to 35 μl min^−1^ and *Q*_G_ was adjusted to give a linear velocity of ~8 mm s^−1^ before reagent addition (measured velocity 8.2 mm s^−1^). The same set-up was used for the two-phase measurements, except the flow-rate of the gas phase was set to zero (*Q*_G_=0), while the reagent phase flow rates *Q*_R1_ and *Q*_R2_ were kept at 30 μl min^−1^, respectively. The flow rate of the carrier phase was adjusted to give a (similar) linear droplet velocity of ~8 mm s^−1^ before reagent addition (measured velocity 8.1 mm s^−1^).

Representative images of the flow before and after the T-junction (that is, before and after reagent addition) are shown in Fig. 4 (see also Supplementary Movies 2 and 3). For the case of the two-phase droplet flow (Fig. 4b,c), a slight irregularity in droplet spacing was visible before entering the T-junction and, on emerging from the junction, the droplets showed significant variations in size, spacing and colouration. Two of the outgoing droplets in Fig. 4c have a deep red colouration, indicating they were newly formed within the T-junction from the freshly introduced red-dyed ODE. The significant size variations and blue-to-purple colouration of the remaining droplets, meanwhile, indicate uneven dosing of the incoming droplets by the red-dyed ODE. (Note, inconsistent dosing was also seen for the case of a two-phase gas-liquid flow, using Ar gas in place of the PFPE carrier fluid—see Supplementary Figure 1 and Supplementary Movie 4.)

In contrast, for the three-phase flow (Fig. 4d,e), the droplets were uniformly sized and spaced both before entering and after leaving the T-junction. Visually, they entered the T-junction with a uniform blue colouration and exited it with a uniform purple colouration, indicating that each droplet had received a similar dose of the red-dyed ODE within the T-junction in accordance with the discussion above.

### Colorimetric analysis of reagent addition

**Figure 5:**
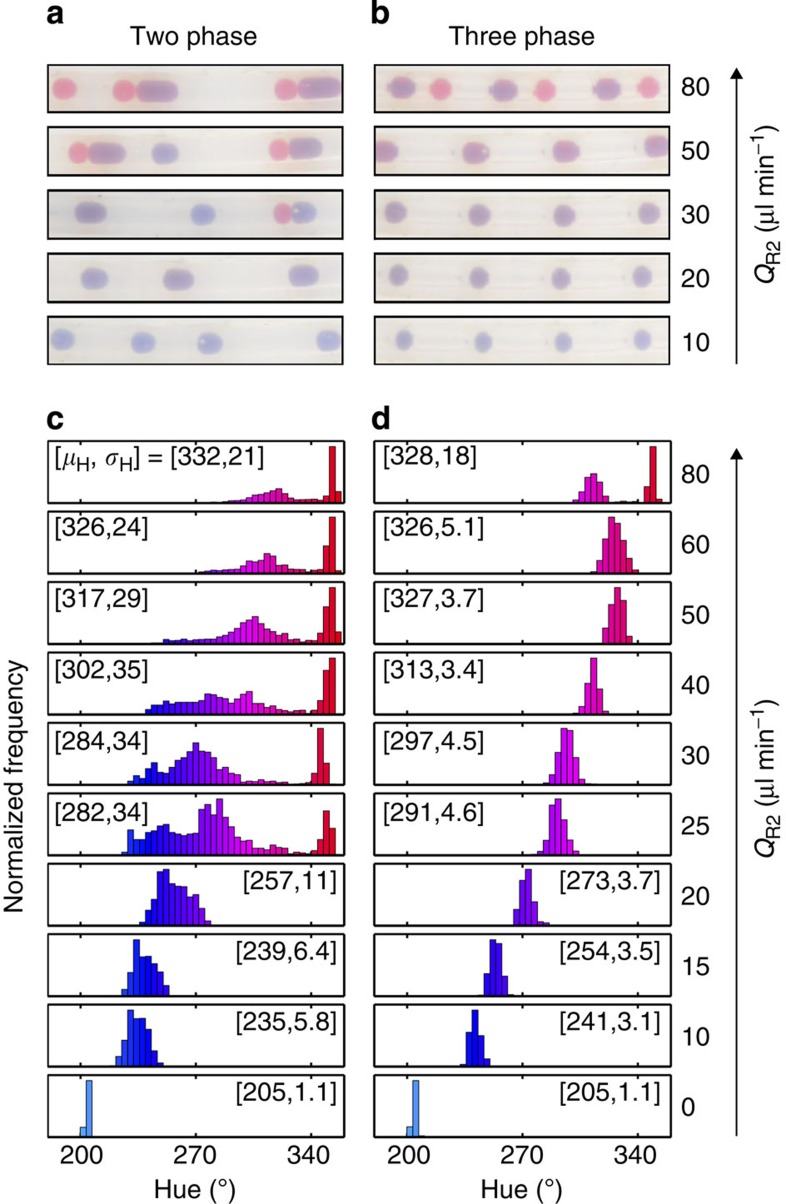
Colorimetric analysis of reagent addition into two- and three-phase fluid streams. (**a**,**b**) Representative images of the flowing droplets after solvent addition for the two- and three-phase flows, under different rates of solvent addition. (**c**,**d**) Hue histograms showing the distribution of droplet colours after solvent addition for different rates of solvent addition. The individual bars of the histogram are shaded with the approximate hue they represent, while *μ*_H_ and *σ*_H_ denote the mean and s.d. of the measured hue, respectively.

The consistency of droplet dosing for the two- and three-phase flows was investigated in more detail by varying the volumetric injection rate *Q*_R2_ of the red dye, while keeping the other flow rates constant. For each value of *Q*_R2_, a movie of the droplet flow downstream of the T-junction was recorded. Representative stills from the movies are shown in Fig. 5a,b. In the case of the liquid/liquid flow (Fig. 5a), the droplet dosing became ever more erratic as *Q*_R2_ was increased from zero, leading to increasing variations in droplet size, spacing and colouration. In the case of the three-phase flow (Fig. 5b), by contrast, the droplets maintained a regular size and colouration until *Q*_R2_ was almost double the value of *Q*_R1_, indicating a far more robust and controlled dosing process.

To quantify the observed behaviour, the hues of the outgoing droplets were determined according to the *Hue, Saturation, Value* (HSV) colorimetry model (see Supplementary Note 1). In the HSV colour scheme, the hue is a single-value parameter that varies from 0 to 360° and indicates the perceived colour of the droplets. Adding increasing amounts of the red-dyed ODE to the blue-dyed ODE droplets causes a progressive change in hue from blue to purple. For the case of highly controlled dosing, in which each blue-dyed ODE droplet receives a near-identical amount of red-dyed ODE, hue values will be distributed tightly around a mean value *μ*_H_, with a small s.d. *σ*_H_. For poorly controlled dosing, droplets with a much broader range of hues will be generated with an increased s.d.

Figure 5c shows a series of hue histograms obtained at different *Q*_R2_ values for the case of two-phase droplet flow, while Fig. 5d shows equivalent data for the case of three-phase flow. When *Q*_R2_ was set equal to zero, that is, when no red-dyed ODE was added to the droplets, narrow and virtually identical distributions were obtained in both cases ([*μ*_H_, *σ*_H_]=[205°, 1.1°]), consistent with all droplets having been generated from the same parent solution of blue-dyed ODE. For the two-phase case, increasing *Q*_R2_ to 10 μl min^−1^ (that is, to one-third the value of *Q*_R1_) resulted in a shift of the mean hue to 235°, consistent with the ‘redder’ nature of the dosed droplets, and an increase in *σ*_H_ to 5.8°. The distribution continued to shift to the right and further broaden as *Q*_R2_ was increased to 15 and then 20 μl min^−1^, with *σ*_H_ increasing to 11° at 20 μl min^−1^. This indicates increasingly erratic dosing of the blue-dyed droplets by the red-dyed ODE as the injection rate of the latter is increased. Indeed for *Q*_R2_ values above 25 μl min^−1^, the dosing was so erratic that the distribution became bimodal, with a new peak emerging at H=350° due to the generation of new droplets containing red-dyed ODE only. Raising *Q*_R2_ still further led to a steady increase in the relative weighting of the 350° peak, indicating an increase in the ratio of red-ODE droplets to mixed droplets, and a further broadening of the mixed droplet peak. Hence, it is clear that, in a conventional two-phase system, a simple T-junction can only be used to achieve reliable dosing of droplets when the volume of added reagent is small compared with the droplet volume.

For the three-phase case, *μ*_H_ shifted smoothly to the right as *Q*_R2_ increased from 10 to 50 μl min^−1^ with *σ*_H_ remaining small (at between 3 and 5°), indicative of uniform dosing of the droplets throughout this range. Only at reagent addition rates above 60 μl min^−1^ (that is, when the injection rate of red-dyed ODE was at least twice that of the blue-dyed ODE) did the downstream addition break down: at 60 μl min^−1^ a slight broadening of the hue histogram to *σ*_H_=5.1° was observed, and at 80 μl min^−1^ a separate peak corresponding to pure red-ODE appeared. Visually this was seen to correspond to discrete slugs of red-ODE having been inserted into the gas spacers, splitting them in two (see Fig. 5b). As can be seen in Fig. 3b and Supplementary Movie 1, each arriving droplet sweeps any accumulated solvent from the tip of the capillary, at which point a new bead of solvent starts to grow. The volume (*V*) of the solvent bead at any point in time is therefore proportional to the time Δ*t* that has elapsed since the departure of the last droplet: *V*(Δ*t*)=*Q*_R2_Δ*t*. The growing solvent bead displaces an equal volume of gas, and in so doing progressively ‘pinches’ the gas spacer (see Fig. 3b, *t*=120 ms). Hence, if the bead grows beyond a critical size V* before the arrival of the next droplet, the gas spacer will be pinched in two, causing a slug of the injected solvent to shear off and be carried away by the separated parts of the gas (as was the case here for *Q*_R2_>60 μl min^−1^). In practice, this problem may be readily avoided by adding the reagent in multiple steps.

### Multistep chemistry in a three-phase reactor

**Figure 6:**
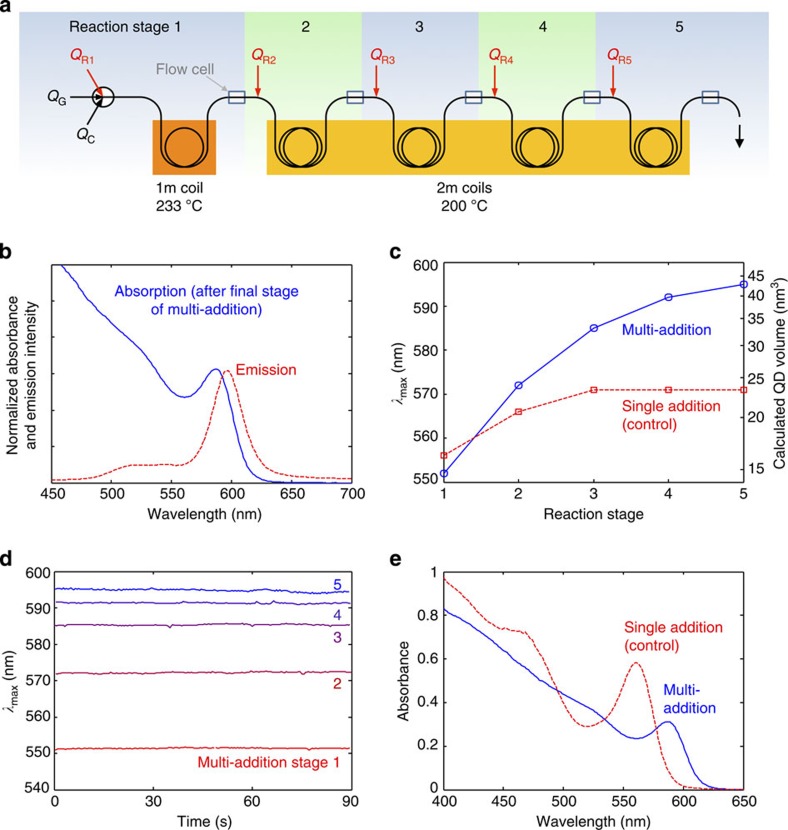
Multistep growth of quantum dots by repeated reagent addition. (**a**) Schematic showing a five-stage reactor used for multistep growth of CdSe quantum dots. (**b**) Normalized absorption and photoluminescence spectra of quantum dots obtained from the reactor following multistep reagent addition; spectra were measured offline using undiluted, unpurified solutions. (**c**) Peak emission wavelength (*λ*_max_) vs reaction stage for multistep and single-step precursor addition; emission spectra were recorded inline using a bifurcated fibre-optic probe, and *λ*_max_ values were determined by averaging over multiple droplets. Indicative particle volumes may be inferred from the emission spectra and are shown on the right-hand *y*-axis (see main text). (**d**) Time-resolved data showing, for each of the five reaction stages during the multi-addition synthesis, the variation in the peak emission wavelength (*λ*_max_) over a 90-s acquisition period. Spectra were acquired inline, and each data point corresponds to a measurement on an individual droplet. (**e**) Absorption spectra of the final as-produced quantum dots for multistep and single-step precursor addition; spectra were measured offline using undiluted, unpurified solutions.

Controlled dosing is a prerequisite for multistep chemistry in droplets. To test the suitability of the three-phase approach for multistep chemical reactions, we applied it to the controlled growth of CdSe quantum dots (QDs) in which additional precursor materials were repeatedly added downstream to replenish the QD feedstock and hence sustain particle growth. The experimental set-up (shown in Fig. 6a) consisted of five separate reaction stages—a high temperature initiation stage (reaction stage one) involving nucleation and initial growth of the QDs followed by four lower temperature growth stages (stages two to five). A flow cell was inserted after each reaction stage so that the progression of the reaction could be monitored by inline fluorescence spectroscopy. Fluorescence spectra were obtained using a bifurcated optical fibre that simultaneously channelled excitation light from a blue laser diode to the droplets and emission light from the droplets to a spectrograph (see Methods).

The initial three-phase flow was generated as described above using a simple three-input conjunction. The reaction phase consisted of a premixed solution of Cd and Se precursors in ODE (see Methods); argon was used as the gas spacer and PFPE as the carrier fluid. The liquid flow rates were set at 60 and 35 μl min^−1^ for *Q*_R1_ and *Q*_C,_ respectively, while *Q*_G_ was adjusted to give a linear velocity after three-phase flow generation of ~8 mm s^−1^ (measured velocity 7.2 mm s^−1^). The three-phase flow was passed into a 1-mm-ID PTFE capillary, one metre of which was immersed in a 233 °C oil bath to initiate nucleation and growth of the quantum dots. After exiting the oil bath, emission spectra of the flowing droplets were recorded in real time as they passed through the first flow cell.

The four subsequent growth stages were carried out in a similar manner. In each case, the three-phase stream first passed through a T-junction where an additional dose of reagent (identical in composition to the initial reaction mixture) was added to the droplets at a rate of 30 μl min^−1^. Next, the droplet stream entered a 2 m length of tubing, which was heated by immersion in a 200 °C oil bath to promote nanoparticle growth. (Note, the growth steps were carried out at a lower temperature than the first reaction stage to avoid fresh nucleation, and the length of heated tubing in stages two to five was increased to allow sufficient time for particle growth). On exiting each oil bath, the three-phase stream passed through a flow cell where emission spectra of the flowing droplets were recorded. After exiting the final flow cell the fluid stream was collected in a beaker, with the reaction phase forming a uniform phase-separated layer above the higher density PFPE carrier fluid. The reaction phase was decanted off, and absorption and emission spectra were recorded (see Fig. 6b). The highly structured absorption spectrum and Gaussian-shaped emission spectrum are consistent with the (expected) formation of CdSe quantum dots.

On exiting each of the four T-junctions, the droplets were visually observed to be of uniform and increased size, consistent with the controlled addition of the new reagent. The emission spectra of the droplets red-shifted progressively with each successive growth stage, with the peak wavelength (*λ*_max_) moving from 552 nm for the freshly nucleated quantum dots (reaction stage one) to a final wavelength of 595 nm after the final growth stage (reaction stage five) as shown in Fig. 6c. Assuming isotropic particles (which are expected for the reagents, concentrations and reaction conditions selected)^9^ and applying the modified Brus Equation proposed by Masumoto^28^, the spectral shift is consistent with a smooth increase in particle diameter from 3.0 nm after the initial nucleation/growth stage to a final diameter of 4.4 nm after the final growth stage, implying a ~2.8-fold increase in particle volume during the ‘growth-only’ stages (see Fig. 6c).

The highly reproducible nature of the reagent dosing is clear from Fig. 6d, which shows the peak emission wavelength obtained from successive droplets over a 90-s acquisition period (see Methods) for each of the five flow cells. Excellent droplet-to-droplet consistency is observed for each flow cell, confirming that identical doses of reagent have been added to each droplet at each stage of the reaction. Indeed, it is striking to note that the droplet-to-droplet consistency at stage five is only marginally worse than that at stage one (that is, before any reagent addition), indicating the potential to carry out many more reagent additions without compromising the consistency of the dosing procedure.

For control purposes, a second experiment was conducted in which the same total amount of reagent was added at the droplet generation stage with no further reagent addition occurring downstream. (that is, *Q*_R1_ was increased to 180 μl min^−1^ while *Q*_R2_ to *Q*_R5_ were set equal to zero). *Q*_C_ and *Q*_G_ remained unchanged. As expected, the linear velocity after 3-phase flow generation increased slightly to 7.8 mm s^−1^. As before, Gaussian emission spectra were measured as the droplets passed through each of the five flow cells, characteristic of successful QD synthesis. This time, however, the shift in the peak wavelength was much smaller. As shown in Fig. 6c, the peak moved progressively from 556 nm after the initial nucleation/growth stage (reaction stage one) to 571 nm after the final heating stage (reaction stage five), implying only a slight increase in diameter from 3.1 to 3.5 nm. The mean particle volume in the case of the single-addition experiment (after the final heating stage) was therefore approximately half that obtained in the case of the multi-addition experiment: ~23 nm^3^ vs ~43 nm^3^ (see Fig. 6c).

In passing, we note that the difference in the final particle volumes correlates well with the difference in particle concentrations deduced by absorption spectroscopy. As shown in Fig. 6e, the single-addition QDs exhibited a much stronger absorption than the multi-addition QDs—indicative of a higher concentration of particles in the former case. Yu *et al.*^29^ have reported an empirical equation relating the molar extinction efficient to the particle size, which in turn can be obtained from the position of the first absorption peak. Using this equation, we determined the particle concentrations to be 24 and 12 μM for the single- and multi-addition processes, respectively. Hence, the data are consistent with both reactions consuming all of the available feedstock but twice as many particles being nucleated in the single-addition procedure, causing the single-step particles to grow to half the final volume of the multistep particles. Hence, to obtain large particles, there is a clear need to dose the droplets repeatedly with small amounts of reagent rather than supply a large upfront dose of feedstock in one go at the point of droplet generation.

## Discussion

The three-phase strategy outlined here provides a straightforward means of implementing a wide range of multistep chemistries in a droplet environment. While the method was used here to grow CdSe quantum dots by repeatedly dosing the droplets with the same precursor material, it should be readily applicable to any procedure falling within the category of ‘one-pot’ synthesis where multiple reagents are added to the same reaction mixture. For example, in the case of quantum dots and other colloidal nanoparticles, it could be utilized to synthesize core/shell structures or to carry out seeded synthesis of anisotropic structures.

In determining whether the three-phase approach will be applicable to a specific multistep chemical procedure, it is necessary to consider the availability of a compatible solvent/carrier/gas triad, the risk of gas evolution during the reaction and the desired production rate. Finding a usable triad will rarely present a significant problem as the pairing of a PFPE carrier fluid with an inert gas carrier should satisfy the vast majority of chemical reactions, except rare cases where some reagents or products are partially soluble in the carrier fluid. Gas evolution is potentially a more significant concern as high rates of gas production can be expected to disrupt the uniformity of the droplet flow and potentially fragment existing droplets. We note, however, that gas spacers have previously been used to ameliorate gas production in flow reactors^30^, with the gas bubbles acting as ‘head-space’ into which the liberated gas is able to gently bleed. In this way, the concentration of dissolved gas in the solvent phase can be kept below the threshold for uncontrolled bubble nucleation, and the uniformity of the flow thereby maintained. Three-phase systems are expected to be similarly tolerant to modest levels of gas evolution, provided the volume of gas emitted per droplet is small in relation to the volume of the gas spacers.

Although so far tested at relatively low flow rates (*Q*_R_<200 μl min^−1^), the method outlined here should scale readily to flow rates of several ml min^−1^. In common with other microfluidic systems, substantially higher levels of throughput are likely to require some form of ‘scale out’ in which the reaction mixture is split into multiple parallel channels. This has previously been achieved for both liquid/liquid^12^ and gas/liquid^31^,^32^ segmented flow reactors. The extension to three-phase systems should be straightforward, with the key challenge being to split each of the incoming carrier, solvent, gas and reagent streams into balanced substreams to ensure matched flow conditions in each channel, and so minimize product variability.

In conclusion we have described a simple, robust strategy for adding controlled amounts of reagent to a flowing stream of droplets. By using a gas phase to maintain an even droplet spacing, simple T-junctions may be used to repeatedly inject new reagent into the droplets at up to double the volume of the existing droplets. The outlined procedure therefore provides a simple means of implementing multistep chemical reactions in droplets.

## Methods

### Three-phase flow generation

The three-phase flow was generated by pumping ODE (Sigma-Aldrich), PFPE (Fomblin Y/LVAC 06/6, Solvay Solexis) and argon gas (Pureshield, BOC) into a simple 3-to-1 flow junction. The liquids were delivered to the junction via fluorinated ethylene propylene (FEP) tubing (Upchurch Scientific, ID 356 μm, OD 1.57 mm) using Braun ‘Injekt’ disposable syringes loaded onto syringe pumps (Harvard Apparatus PHD 2000 and Pump 11+ models). The syringes were connected to the tubing using polyether ether ketone Luer-Lock interconnects (Upchurch Scientific). Argon gas was delivered to the junction from a gas cylinder via fused silica capillary (CM Scientific, ID 150 μm, OD 375 μm), with the flow rate regulated by a mass flow controller (Sierra Instruments MicroTrak 101).

The 3-to-1 flow junction was fabricated in-house using a 4-dimensional CNC mill to machine PTFE round stock. As shown in Supplementary Fig. 3, the junction had 1 mm through-channels and was interfaced with tubing using flangeless ferrules and nuts (P-305, P-342, Upchurch Scientific). The three-phase flow exited the junction into PTFE tubing (Polyfon, ID 1 mm, OD 2 mm).

### T-junction fabrication

T-junctions were custom fabricated in optically transparent FEP tubing to enable observation of the solvent/reagent addition (see Supplementary Fig. 4). The junctions comprised a 1-mm ID main channel for the three-phase flow and a smaller (150 μm ID) side channel for delivery of the new solvent/reagent. The T-junctions were fabricated as follows:

A 400 μm hole was drilled into one wall of a ~5 cm length of transparent FEP tubing (Polyflon, ID 1 mm, OD 2 mm), FEP being chosen for its fluorous surface and optical transparency. A 5 cm length of fused silica capillary (CM Scientific, ID 150 μm, OD 375 μm) was partially inserted into the hole, taking care to ensure it did not intrude into the channel itself, and then fixed in place using a small quantity of epoxy (Araldite 2014, Huntsman). To make the junction permanent, the entire structure was encased in a solid slab of polydimethylsiloxane by creating a mould around the T-junction with putty (leaving the free ends of the tubing exposed), pouring liquid polydimethylsiloxane (Sylgard 184) into the mould, degassing under vacuum and then curing in a 60 °C oven overnight. After removal of the mould, the junction was ready to be used (see Supplementary Fig. 4). The free ends of the FEP tubing and silica capillary were joined to PTFE tubing using silicone tubing (VWR, ID 1 mm, OD 3 mm).

### Analysis of droplet flow

Movies of the droplet flow were acquired using a colour digital single-lens reflex (DSLR) camera (Canon EOS 550D) fitted with a 60 mm macro USM lens (Canon EF-S 60 mm). The linear velocity of the fluid stream was determined using droplet tracking software (Droplet Morphometry and Velocimetry by Amar Basu^33^). The colorimetric characteristics of the droplets were determined from the movies using Matlab scripts developed in-house (Supplementary Note 1 for a discussion of colorimetry methods used).

### Quantum dot synthesis

The PFPE carrier fluid (Galden HT270, Solvay Solexis) was obtained from Kurt J. Lesker. All other chemicals were obtained from Sigma-Aldrich.

The Cd precursor solution was made as follows: 520 mg of CdO (99.99+ %), 24 ml of oleic acid (90%) and 400 ml of ODE (90%) were added to a 500-ml round bottom flask. The mixture was degassed under vacuum, left under an argon atmosphere, and then heated by immersion in an oil bath to ~200 °C, at which point the mixture clarified to become a colourless solution. The solution was then allowed to cool to room temperature before use.

The Se precursor solution was prepared by adding 600 mg of Se powder (99.5+ %), 32 ml of trioctylphosphine (95%) and 400 ml ODE to a 500 ml round bottom flask, degassing under vacuum, and then stirring at room temperature under an argon atmosphere until all the Se had dissolved.

Equal volumes of the Cd and Se precursor solutions were combined to yield the CdSe precursor solution.

### Offline optical characterization

Offline photoluminescence and absorption spectra of the undiluted, unpurified product were obtained using a Jobin-Yvon Fluoromax-2 fluorimeter. A photodiode in the fluorimeter was used to monitor the transmitted light through the cuvettes to obtain absorption spectra.

### Inline fluorescence spectroscopy

Inline fluorescence spectra were obtained using five custom-built flow-cells that were probed in sequence using a bifurcated fibre-optic (Ocean Optics). Each flow cell comprised transparent FEP tubing that passed through the central axis of an opaque cylindrical casing with an orthogonal access port for the fibre-optic probe (see schematic shown in Supplementary Fig. 5). The cylindrical casing was fabricated by drilling out 2 cm diameter black Delrin round-stock (RS Components). The bifurcated fibre-optic probe was successively inserted into each access port of the five flow-cells and used to channel excitation light from a blue (405 nm) laser diode to the probe volume and emitted light from the flowing droplets to a CCD spectrometer (Ocean Optics USB 2000).

## Author contributions

A.M.N. conceived the three-phase strategy for reagent addition. A.M.N., T.W.P. and J.H.B. developed and tested the experimental set-up. A.M.N. carried out the experiments. A.M.N. and J.C.deM. designed the experiments and wrote the manuscript.

## Additional information

**How to cite this article:** Nightingale, A. M. *et al.* Controlled multistep synthesis in a three-phase droplet reactor. *Nat. Commun.* 5:3777 doi:10.1038/ncomms4777 (2014).

## Supplementary Material

Supplementary Figures, Note and Reference Supplementary Figures 1-5, Supplementary Note 1 and Supplementary Reference

Supplementary Movie 1 Movie showing the direct injection of a laminar stream of red-dyed octadecene into a three-phase fluid stream, consisting of droplets of blue-dyed octadecene and bubbles of Ar within a perfluorinated polyether carrier liquid.

Supplementary Movie 2 Movie showing a two-phase liquid/liquid stream (consisting of droplets of blue-dyed octadecene within a perfluorinated polyether carrier liquid) before and after the introduction of a laminar stream of red-dyed octadecene.

Supplementary Movie 3 Movie showing a three-phase fluid stream (consisting of droplets of blue-dyed octadecene and bubbles of Ar gas within a perfluorinated polyether carrier liquid) before and after the introduction of a laminar stream of red-dyed octadecene.

Supplementary Movie 4 Movie showing a two-phase gas/liquid stream (consisting of slugs of blue-dyed octadecene separated by Ar gas) before and after the introduction of a laminar stream of red-dyed octadecene.

## Figures and Tables

**Figure 1:**
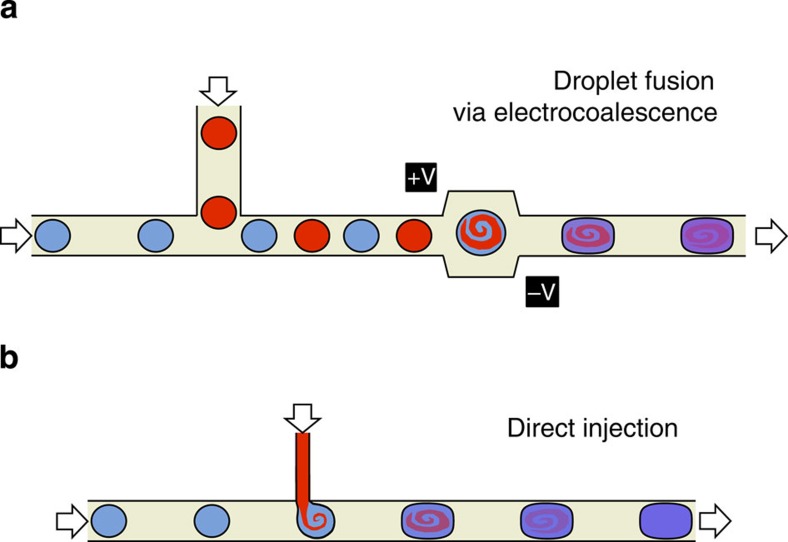
Reagent addition in droplet flow. Image showing the two principal methods for adding additional solvent/reagent to a flowing droplet stream: (**a**) droplet fusion, in which a second droplet stream containing the new solvent/reagent is merged on a pairwise basis with the original droplet stream, using an applied voltage or specially engineered channel structure^18^; and (**b**) direct injection, in which the new solvent/reagent is introduced as a continuous stream that directly inserts into the droplets.
