# Supplementary material for: Controlled multistep synthesis in a three-phase droplet reactor
Source: Nat Commun. 2014 May 6;5:3777. doi: 10.1038/ncomms4777 (PMC4024758; doi:10.1038/ncomms4777)
Supplement: Supplementary Figures, Note and Reference — Supplementary Figures 1-5, Supplementary Note 1 and Supplementary Reference [file ncomms4777-s1.pdf]

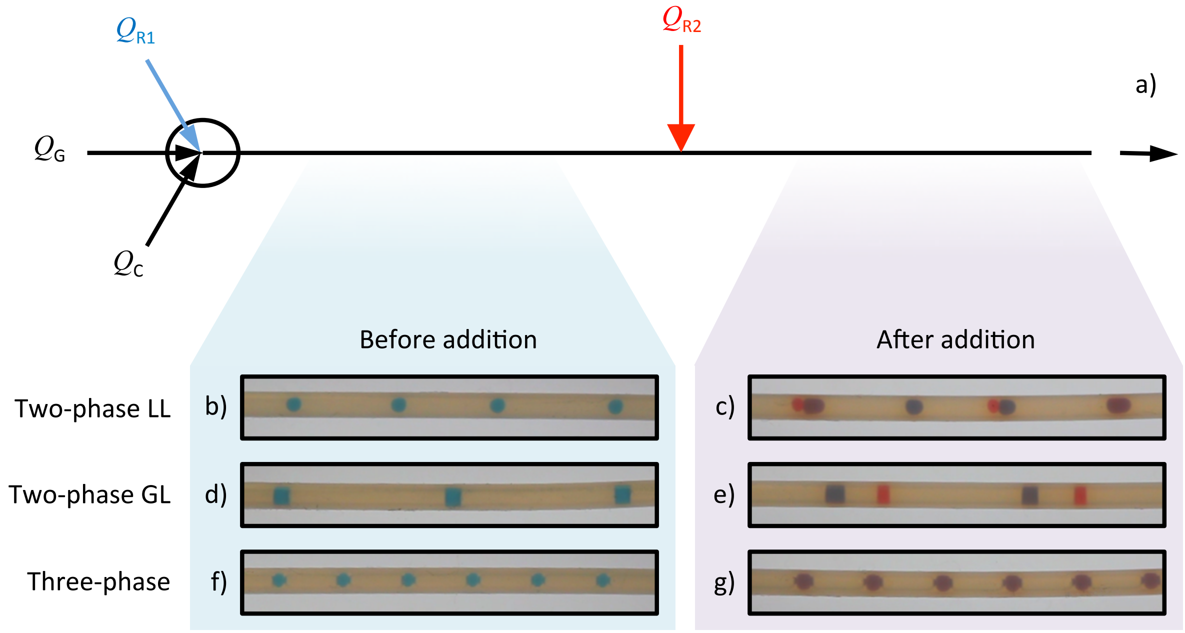

Supplementary Figure 1 | Droplet flow before and after solvent addition. (a) Schematic showing experimental setup used to observe the injection of red-dyed ODE into blue-dyed slugs or droplets of ODE: a two- or three-phase stream of blue-dyed ODE slugs/droplets is first generated by combining ODE/PFPE, ODE/Ar or ODE/PFPE/Ar in a passive mixer, and a stream of red-dyed ODE is then injected at a rate Q_{R2} into the flowing droplets using a T-junction. (b-g) Representative images of the flowing droplets before and after solvent addition for the liquid/liquid (LL), gas/liquid (GL) and gas/liquid/liquid (three-phase) flows.

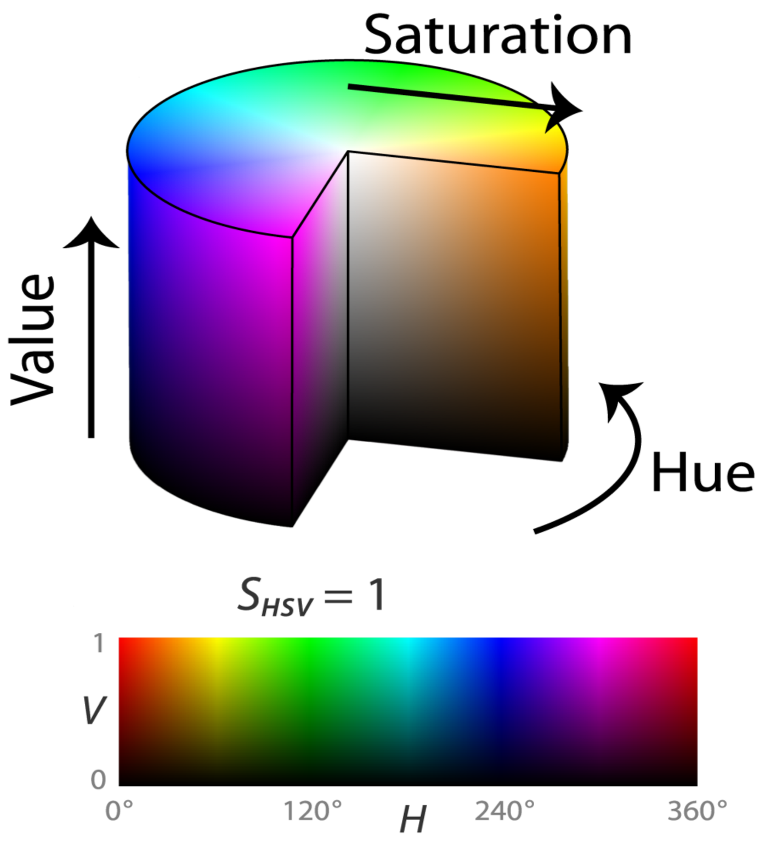

Supplementary Figure 2 | HSV colour space. A three-dimensional cut-away representation of the Hue-Saturation-Value (HSV) colour space, plus a two-dimensional plot in which S is held constant while H and V are varied. Image modified from "Hsl-hsv models.svg" by Jacob Rus. This file is licensed under the Creative Commons Attribution-Share Alike 3.0 Unported licence (http://creativecommons.org/licenses/by-sa/3.0), via Wikimedia Commons.

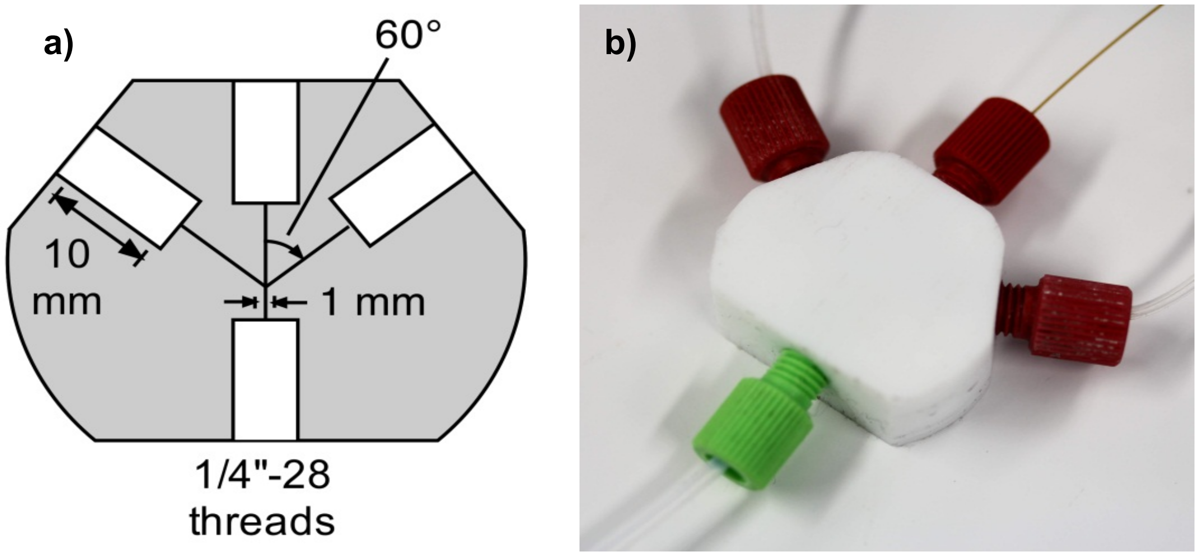

Supplementary Figure 3 | Structure of droplet generators. Schematic (a) and photograph (b) of a micro-machined PTFE junction used for generating two- and three-phase flows. Two-phase flows were generated by setting the flow rate of the unwanted fluid to zero.

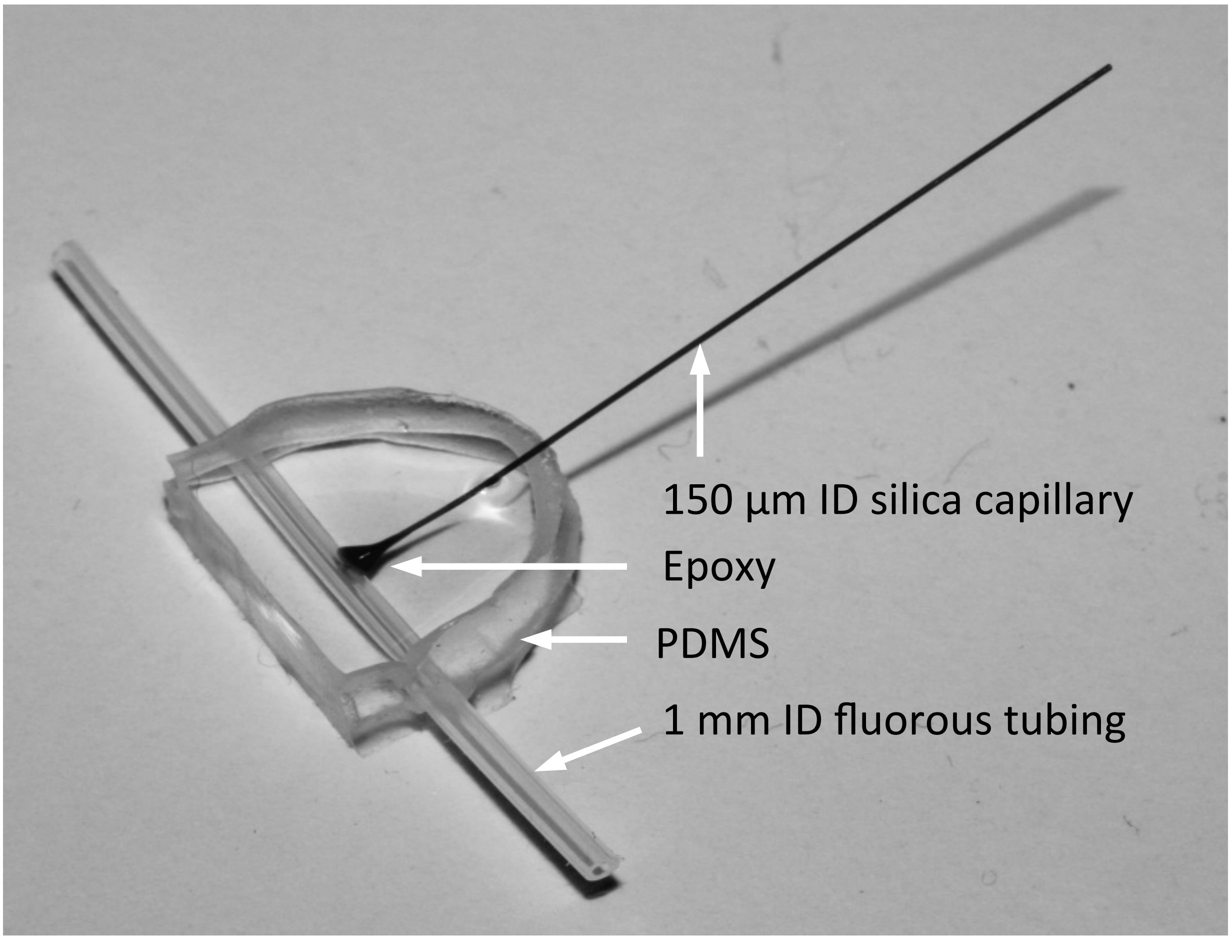

Supplementary Figure 4 | T-junction used for reagent addition. Photograph of a T-junction, comprising an FEP main channel though which the droplet stream flows and a silica side channel for injecting additional reagent. The T-junction is embedded in a slab of PDMS for support.

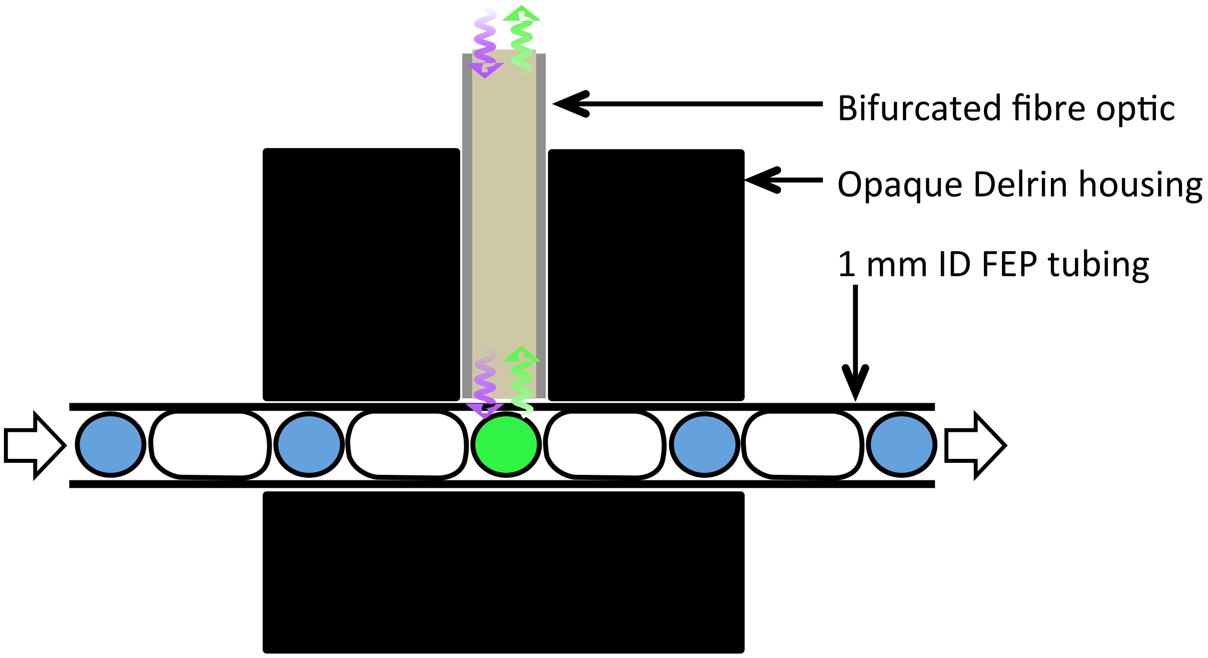

Supplementary Figure 5 | Schematic of flow-cell. The flow-cells comprise a transparent 1 mm inner diameter FEP tube threaded through the centre of an opaque Delrin cylinder, into which is drilled an orthogonal access port to allow insertion of a bifurcated fibre-optic. The fibre optic is used to channel excitation light from a 405 nm LED to the probe volume and to channel emitted light from the probe volume to a CCD spectrometer.

Supplementary Note 1 | HSV Colour Space

In the *Hue, Saturation, Value* (HSV) colorimetry model, colour is represented as a cylindrical polar coordinate (see Supplementary Figure S2). The azimuthal angle, which varies from 0 to 360°, denotes the hue (or perceived colour) of the object; the radial coordinate, which varies from 0 to 1, denotes its saturation (or colour purity); and the axial coordinate denotes its value (or brightness).

The hue (*H*) of individual droplets was obtained by taking RGB data (averaged from multiple pixels in the droplet) from movie stills and applying Supplementary Equation 1:[1]

$$H = \tan^{-1}\frac{\sqrt{3}(G-B)}{2R-G-B} \tag{1}$$

Supplementary Reference

1 Hanbury, A. Constructing cylindrical coordinate colour spaces. *Pattern Recognition Letters* **29**, 494-500, doi:10.1016/j.patrec.2007.11.002 (2008).
